# Organochlorine Exposure and Colorectal Cancer Risk

**DOI:** 10.1289/ehp.7143

**Published:** 2004-07-15

**Authors:** Mike Howsam, Joan O. Grimalt, Elisabet Guinó, Matilde Navarro, Juan Martí-Ragué, Miguel A. Peinado, Gabriel Capellá, Victor Moreno, for the Bellvitge Colorectal Cancer Group

**Affiliations:** ^1^Laboratoire Universitaire de Médécine du Travail, Lille, France; ^2^Consejo Superior de Investigaciones Cientificas, Department of Environmental Chemistry, Institute of Chemical and Environmental Research, Barcelona, Catalonia, Spain; ^3^Catalan Institute of Oncology, Barcelona, Catalonia, Spain; ^4^Ciudad Sanitaria i Universitaria de Bellvitge, University of Barcelona, Barcelona, Catalonia, Spain; ^5^Oncology Research Institute, Barcelona, Catalonia, Spain

**Keywords:** case–control study, colorectal cancer, K-*ras* mutations, organochlorines, *p53* mutations, PCBs

## Abstract

Organochlorine compounds have been linked to increased risk of several cancers. Despite reductions in their use and fugitive release, they remain one of the most important groups of persistent pollutants to which humans are exposed, primarily through dietary intake. We designed a case–control study to assess the risk of colorectal cancer with exposure to these chemicals, and their potential interactions with genetic alterations in the tumors. A subsample of cases (*n* = 132) and hospital controls (*n* = 76) was selected from a larger case–control study in Barcelona, Catalonia, Spain. We measured concentrations in serum of several organochlorines by gas chromatography. We assessed point mutations in K-*ras* and *p53* genes in tissue samples by polymerase chain reaction/single-strand conformation polymorphism and assessed expression of p53 protein by immunohistochemical methods. An elevated risk of colorectal cancer was associated with higher serum concentrations of mono-*ortho* polychlorinated biphenyl (PCB) congeners 28 and 118. The odds ratio for these mono-*ortho* PCBs for middle and higher tertile were, respectively, 1.82 [95% confidence interval (CI), 0.90–3.70] and 2.94 (95% CI, 1.39–6.20). α-Hexachlorocyclohexane, hexachlorobenzene, and *p*,*p*′-DDE (4,4′-dichlorodiphenyltrichloroethene) showed nonsignificant increases in risk. Risk associated with mono-*ortho* PCBs was slightly higher for tumors with mutations in the *p53* gene but was not modified by mutations in K-*ras*. Mono-*ortho* PCBs were further associated with transversion-type mutations in both genes. These results generate the hypothesis that exposure to mono-*ortho* PCBs contributes to human colorectal cancer development. The trend and magnitude of the association, as well as the observation of a molecular fingerprint in tumors, raise the possibility that this finding may be causal.

Colorectal cancer is the third most common human cancer and the second most important cause of cancer-related death in Western countries, affecting men and women about equally. The etiology of sporadic colorectal cancer is relatively poorly understood, although diet is thought to play an important role in modifying risk. Vegetables, fruit, and dietary fiber are protective, whereas red and processed meats, fat, total energy intake, and obesity all increase risk (Potter 1996).

Diet is also an important source of exposure to many synthetic organic chemicals used in industry, agriculture, or accidentally released to the environment. Among them, the industrial organochlorine compounds (OCs) hexachlorobenzene (HCB) and polychlorinated biphenyls (PCBs) and the pesticides DDT (dichlorodiphenyltrichloroethane) and lindane [γ-hexachlorocyclohexane (HCH), including α-HCH and β-HCH isomers] have been classified as “probably” or “possibly” carcinogenic to humans [International Agency for Research on Cancer (IARC) 1987, 1991]. Most of the data for these classifications come from animal studies, although evidence for carcinogenic effects in humans is accumulating from occupational and nonoccupational exposure studies [Agency for Toxic Substances and Disease Registry (ATSDR) 2000, 2002].

Despite reductions in their use and fugitive release, OCs remain one of the most important groups of persistent pollutants to which humans are exposed, primarily via dietary intake. More lipophilic OCs, and those that are not easily metabolized, accumulate in adipose tissue, and the half-lives of these compounds in the body can be on the order of years or decades, whereas those compounds that are more water soluble or more easily metabolized have half-lives on the order of hours or days. Eventually, OCs recirculate in blood and are excreted in feces (Moser and McLachlan 2001). Serum concentrations are strongly correlated with fecal concentrations, particularly for the more lipophilic compounds that accumulate in adipose tissue and are generally more chlorinated (Juan et al. 2002; Moser and McLachlan 2001). Exposure of tissue in the gastrointestinal tract to OCs is the result, therefore, not only of uptake from food but also of depuration from the tissue to the lumen. The long residence time (1–2 days) of feces in the colon offers potentially greater opportunity for exchange of OCs between the lumen and the epithelium than elsewhere in the gastrointestinal tract. However, the physicochemical characteristics of the compound (specifically, its solubility in water) will be more important in determining the relative importance of these exchange processes in the colon than in the small intestine, given the predominantly aqueous nature of the colonic milieu (Moser and McLachlan 2001; Schlummer et al. 1998). Therefore, colon epithelium is likely to be a major target for putative carcinogenic effects of OCs via luminal and blood-borne exposure.

OCs have been shown to mimic hormones, and this has been postulated as a mechanism for carcinogenesis in hormone-dependent cancers (Davis et al. 1993). Although colorectal cancer cannot be considered a hormone-dependent cancer, there is evidence that hormones play a role, at least in women: hormone replacement therapy and, possibly, high parity and oral contraceptive use are all protective factors (Potter 1999). Studies of cancers of the pancreas and breast have shown that OCs may interact with genetic alterations in tumors such as K-*ras* mutations or p53 overexpression (Hoyer et al. 2002; Porta et al. 1999; Slebos et al. 2000). Research on these interactions is relevant because they are frequent in colorectal cancer, and one potential mechanism of OC toxicity may be the induction of mutations in these genes.

Despite this, few studies of OC exposure and colorectal cancer risk have been published. Some studies in occupationally exposed cohorts have shown mixed results (Acquavella et al. 1996; Bertazzi et al. 1987; Brown 1987; Cantor and Silberman 1999; Leet et al. 1996; Wilkinson et al. 1997), whereas studies with data on individual plasma levels are rare. One study of colorectal cancer in children from rural areas exposed to pesticides showed no differences in serum OC levels compared with controls (Caldwell et al. 1981). Higher (nonsignificant) serum concentrations of DDT and β-HCH in cases than in controls were reported in a small study of farmers in Egypt (Soliman et al. 1997).

To test the hypothesis that exposure to OCs plays an etiologic role in colorectal cancer, we determined serum concentrations in a subset of samples from a hospital-based case–control study. In addition, we investigated the relationship between OC concentrations and mutations of the K-*ras* and *p53* genes.

## Materials and Methods

### Study population.

We conducted a hospital-based case–control study to assess gene–environment interactions in relation to colorectal cancer risk. A detailed description of the population and study methods has already been published (Landi et al. 2003). Cases were patients with a new diagnosis of colorectal adenocarcinoma attending a university hospital in Barcelona, Catalonia, Spain, between January 1996 and December 1998. Controls were selected from admissions to the same hospital during this period using age and sex as frequency-matching criteria. To avoid selection bias of controls, the reason for admission had to be a disease not previously diagnosed for that patient. This criterion was used to avoid inclusion of patients with chronic diseases who might be repeatedly admitted to hospital and modify their habits because of their disease. This procedure paralleled the criterion for cases, who were also newly diagnosed incident cases. Main diagnosis groups of controls were acute digestive surgery (19%), urology (17%), gastroenterology (16%), and orthopedic surgery (15%). The study protocol was cleared by the ethics committee of the hospital, and all individuals gave written, informed consent to participate and for genetic analysis of their samples to be performed.

Of 523 identified cases, there were 13 (2%) refusals and 74 (14%) exclusions, with reasons including mental or other impairment and death or discharge before recruitment. We identified a total of 488 controls, with 36 (7%) refusals and 22 (5%) exclusions (reasons as described above). From these 436 cases and 430 controls, we selected a random sample of 140 cases and 80 controls for OC analyses for budgetary reasons. The sample size and imbalance in number between cases and controls was chosen to retain statistical power for our study of K-*ras* and *p53* mutations among cases [80% power to detect odds ratios (ORs) of 2.25 for the exposures of interest]. Controls for this study were selected using strata based on age, sex, and energy intake, the latter being used as a frequency-matching criterion because diet is a main source of exposure to OCs. Twelve samples were lost or excluded because of bad quality during laboratory analyses, and statistical tests were conducted on 132 cases and 76 controls.

### Interviews.

Cases and controls were interviewed by trained personnel using a structured questionnaire. A dietary history questionnaire, previously developed and validated in the framework of the EPIC study [European Prospective Investigation into Cancer and Nutrition (EPIC) Group of Spain 1997], focused on average food consumption 1 year before diagnosis. Food groups based on bromatologic properties were calculated from reports of items consumed. Other risk factors measured were body mass index (BMI) at diagnosis and 10 years before, parity in women, and life-long history of nonsteroidal anti-inflammatory drugs (NSAIDs), tobacco, and alcohol use.

### Organochlorine analysis.

Selection of OCs for analysis was based on a literature search of commonly studied contaminants and included representatives of industrial and agrochemical compounds. Among the large family of PCB congeners, those often referred to as the ICES 7 (from International Council for the Exploration of the Sea), were selected: mono-*ortho* PCB congeners 28 and 118 and di-*ortho* PCBs 52, 101, 138, 153, 180. These PCB congeners are frequently found at high concentrations in humans and wildlife.

Nonfasting blood samples were obtained at diagnosis, and serum was isolated and analyzed “blind” for OCs. We calculated lipid-corrected concentrations of OCs by dividing serum OC concentrations by total plasma lipid concentrations estimated using the formula of Phillips et al. (1989), and data are expressed in nanograms of OC per gram of lipid.

OCs were analyzed by gas chromatography after serum samples were subjected to liquid–liquid extraction and cleanup using concentrated sulfuric acid and hexane (Porta et al. 1999). A mass spectrometer in negative chemical ionization mode was used to quantify α-, β-, γ-, and δ-HCH isomers; pentachlorobenzene (Pe-CB), HCB, PCB congeners, *p*,*p*′-DDT, and *p*,*p*′-DDE (4,4′-dichlorodiphenyltrichloroethene) were quantified on a gas chromatograph with electron capture detection. On both instruments, quantification was performed by external standards using PCB-142 as an injection standard to correct for volume. This method performed satisfactorily in an international intercalibration exercise within the Arctic Monitoring and Assessment Programme (AMAP 2004).

### Point mutations in K-ras and p53 genes, and expression of p53 protein.

Fresh tumor tissue and normal mucosa samples were obtained from surgically extracted specimens of cases. K-*ras* gene mutations in codons 12 and 13 were detected and characterized by polymerase chain reaction (PCR) followed by single-strand conformation polymorphism (SSCP) analysis (Tortola et al. 1999). Aspartic acid mutations at codons 12 and 13 were confirmed by means of artificially introduced restriction fragment length polymorphism. Similarly, *p53* exons 4–9 were analyzed by PCR and SSCP followed by direct sequencing whenever necessary. Expression of the p53 protein was determined by immunohistochemistry, using commercially available antibodies (ab-6 Pantropic; Oncogene Research Products, VWR International GmBH, Darmstadt, Germany). We studied microsatellite instability by analyzing five microsatellite sequences (Gonzalez-Garcia et al. 2000).

### Statistical analysis.

OC concentrations were categorized using tertiles based on all subjects. Some compounds were not detected in a high proportion of individuals. For these, the reference category consisted of values below the detection limit (LOD). When the proportion of individuals with detectable values was > 50%, the values were further divided into two groups using the median value of those with values > LOD.

Logistic regression was used to test the association between OCs and colorectal cancer. When cases were subdivided into groups according to genetic alterations, we used polytomous logistic regression, comparing each group of cases with the whole set of controls. All analyses were adjusted for age, sex, total energy intake, and BMI at diagnosis because these had been used as frequency-matching criteria or, in case of BMI, because of concerns about potential confounding of OC exposure by BMI, given that cases showing lower BMI might have undergone lipid-stored OC mobilization. The impact of these adjustments on risk estimates was minimal, however. We also excluded an association between OCs and tumor stage that would be indicative of bias, because more advanced tumors are associated with greater weight loss. Potential confounding by other variables associated with disease (alcohol, NSAIDs, and food groups) was explored and rejected. These were not included in the models to avoid losing efficiency in the estimates owing to excessive stratification.

ORs and 95% confidence intervals (CIs) were calculated for each group compared with the reference category. We tested for linear trend using the categorized variable as quantitative after assigning codes 1 to 3 to each category defined by tertiles. Interactions between exposures and genetic alterations in tumors were tested with logistic regression models comparing cases with the alteration to cases without it. All *p*-values calculated were two sided.

## Results

Characteristics of cases and controls are shown in Table 1. Sex, age, and energy intake were used as frequency-matching criteria. BMI estimated 10 years before diagnosis was not related to disease. In contrast, BMI at diagnosis was inversely related to disease, probably due to cancer-related weight loss, although no association was found between plasma lipids and disease, or between plasma lipids and OC concentrations. In this population, alcohol intake, but not smoking, was a strong risk factor for colorectal cancer, both in duration and in average daily consumption. NSAIDs other than aspirin were protective but not significantly so.

### OC levels.

Lipid-corrected concentrations of OCs are shown in Table 2. The most abundant compound was *p*,*p*′-DDE, followed by HCB and β-HCH. Summed PCB concentrations were similar to those of β-HCH. The proportions of samples with values lower than LODs are also shown in Table 2. PCB-28 and PCB-52 were detected in only a small proportion of cases, although the LOD was very low. These compounds are more easily metabolized than are other PCBs because of the arrangement of their chlorine atoms and their lower degree of chlorination (Borlakoglu and Walker 1989). δ-HCH was not detected in any individual, and although Pe-CB and PCB-101 were detected in a few samples, their LODs were higher than other OCs; these three compounds were therefore excluded from statistical analyses.

Compounds were divided into two groups according to water solubility, and cases had slightly higher levels than did controls of both groups (data not shown). PCBs were grouped according to their structure, which is related to toxicity (ATSDR 2000; Safe 1990). Mono-*ortho* PCBs 28 and 118 have a relatively “planar” configuration compared with the remaining, di-*ortho* PCBs measured here. Exposure to mono-*ortho* PCBs 28 and 118 was almost double in cases compared with controls, whereas exposure to di-*ortho* PCBs was only slightly higher for cases than for controls.

### OC levels and colorectal cancer risk.

Table 3 shows median lipid-corrected concentrations and 5th and 95th percentiles for each compound, separated for cases and controls. ORs for the association of OCs with colorectal cancer, adjusted for age, sex, BMI, and energy intake, are shown in Table 4. Those significantly associated with an increased risk were mono-*ortho* PCBs 28 and 118, each with individual ORs > 2 for the more exposed category. The OR for serum concentrations of the mono-*ortho* PCB group that combines PCB-28 and PCB-118 was 1.82 (95% CI, 0.90–3.70) in the middle tertile (LOD, 147 ng/g lipid) and 2.94 (95% CI, 1.39–6.20; *p*-value for trend = 0.004) for the highest tertile. Concentrations of di-*ortho* PCBs were not related to disease, nor was the classification of OCs according to their lipophilicity. High levels of α-HCH, HCB, and *p*,*p*′-DDE showed a nonsignificant increase in risk. Levels of *p*,*p*′-DDT were lower in cases than in controls in this population.

### OC levels and interaction with other variables.

Dietary variables, which are both sources of exposure and risk factors for colorectal cancer, were explored as potential confounders. Specific food groups explored were vegetables, fruit, legumes, potatoes, meat, fish, eggs, dairy products, added fats, and pastries. Spearman’s correlation coefficients between food groups and serum levels of specific OCs were in general low and nonsignificant (maximum *r* = 0.19). Exceptions worth mentioning were dairy products, which correlated with *p*,*p*′-DDT and PCB-101; fresh meats, which correlated with PCB-101, PCB-153, and PCB-180; and fish, which correlated with PCB-153 and PCB-180. The significance of these correlations was poor, and in addition to those correlations mentioned, we observed several negative correlations. We also used a multivariate logistic regression model with all food groups to generate a “dietary propensity factor,” which could be interpreted as a score integrating the risk of colorectal cancer associated with diet. Adjusting OCs for this dietary propensity factor did not modify the risk estimates, showing again that diet was not a confounding factor. Hence, after careful consideration, adjustment of the analyses for food groups was found to be unnecessary because foods were not clearly related to serum OC levels and, more important, did not modify the risk estimates.

An exploratory analysis of interactions between mono-*ortho* PCBs and several variables was carried out. The magnitude of the risk of colorectal cancer observed for these OCs was not modified by sex, age, BMI, smoking, NSAIDs use, or parity in women. Analysis of interactions with other hormonal variables in women could not be performed because only six women were exposed to oral contraceptives and only two to postmenopausal hormone replacement treatments. Because alcohol was an important risk factor in this study and alcohol intake produces metabolic induction similar to that produced by some OCs (Mochizuki and Yoshida 1989), interactions between OCs and alcohol were explored in detail, but none was significant.

### OC levels and mutations at the K-ras and p53 genes.

K-*ras* was mutated in 50 (38%) cases. Most frequent mutations were GAT (*n* = 16) and GTT (*n* = 15) in codon 12. In 6 cases, codon 13 was mutated to GAC. Other, less frequent mutations in codon 12 were TGT (*n* = 5), AGT (*n* = 4), GCT (*n* = 3), and CGT (*n* = 1). Twenty-six mutations were transitions (G:C → A:T), and 24 were transversions (G:C → T:A).

Tumor suppressor gene *p53* was mutated in 59 (60%) of the tumors analyzed (data not available for 34 cases). Twenty-four mutations were located in hotspots: codon 175 (*n* = 4), codon 245 (*n* = 3), codon 248 (*n* = 3), codon 273 (*n* = 6), and codon 282 (*n* = 8). Four cases harbored mutations at codon 158, the remaining being detected at 24 different codons with frequencies ≤ 2. Forty mutations were transitions, and 15 were transversions; in the remaining four cases, deletions were detected. The most frequent base changes were C → T and G → A, each in 17 cases, mainly at hotspot sites. G → T changes were observed in eight cases and distributed with a nonclustered profile. The immunohistochemical analyses showed that 76% of the cases overexpressed the p53 protein.

Table 5 shows the analysis of selected OCs where the cases have been stratified by mutations in K-*ras* and *p53*. This analysis is based on polytomous logistic regression, and each group of cases is compared with controls in a unified model. Exposure to mono-*ortho* PCBs 28 and 118 increased risk in similar magnitude for both mutated and wild-type K-*ras*. PCB-118, but not PCB-28, showed higher risk for *p53*-mutated tumors, although the interaction was not significant. This stratification of cases by genetic alterations also showed some significant interactions with other OCs. High levels of *p*,*p*′-DDE were associated with increased risk of cancer with wild-type K-*ras*. Also, *p*,*p*′-DDE and α-HCH had significant effects on *p53*-mutated tumors. A similar pattern was evident when tumors were stratified according to p53 overexpression: *p*,*p*′-DDE and PCB-118 increased the risk only for tumors overexpressing p53 protein. Tumors with microsatellite instability were rare (*n* = 11, 8%), and these patients had similar OC levels to other cases.

Overall, 81% of the tumors harbored K-*ras* and/or *p53* gene mutations, and a combined analysis was performed. Exposure to high levels of the mono-*ortho* PCB-118 was associated with more mutations (OR = 2.89; 95% CI, 0.66–12.7), with transversions three times more likely than transitions (Table 6). Other OCs analyzed in this way were not related to the type of mutation.

## Discussion

OCs have previously been associated with increased risk of colorectal cancers in studies of occupationally exposed individuals (Acquavella et al. 1996; Soliman et al. 1997; Wilkinson et al. 1997). Other studies have failed to show this association (Hardell 1981; Hoar et al. 1985; Settimi et al. 2001) or even have shown inverse associations with colon cancer (Cantor and Silberman 1999; Wang et al. 2002). These mixed results are not surprising because most occupational studies lack individual exposure indicators and are prone to confounding. To overcome these limitations, we studied a population not occupationally exposed using serum OC levels as exposure markers. We found an elevated risk of colorectal cancer associated with high levels of mono-*ortho* PCBs 28 and 118. Other abundant OCs such as *p*,*p*′-DDE and α-HCH also showed an increased risk of relevant magnitude; although the associations for these OCs were not significant for the entire population of cases, they were significant for the subset of tumors harboring mutations of the *p53* gene.

We have taken serum concentrations of OCs as indicative of the body burden. Serum concentrations are strongly correlated with those in adipose tissue and feces and reflect the historical legacy of uptake and depuration, and as such, they may be taken as markers for long-term, low-level exposure (Alcock et al. 2000; Juan et al. 2002; Moser and McLachlan 2001). Many studies that also used plasma OC concentrations as exposure markers have reported mixed results for breast cancer (Calle et al. 2002) and non-Hodgkin lymphoma (Cantor et al. 2003; De Roos et al. 2003; Rothman et al. 1997) but increased risk of pancreatic cancer (Hoppin et al. 2000; Porta et al. 1999; Slebos et al. 2000). Altogether, these results suggest that a role for OCs in the etiology of several tumors is likely. Nevertheless, the mechanisms underlying carcinogenicity will differ among both OCs and target organs because chemical properties and toxicity mechanisms of these compounds are diverse. In addition, target organs differing in lipid content might determine distinct degree of local exposure.

In our population, high body burdens of mono-*ortho* PCBs 28 and 118 were associated with an elevated risk of colorectal cancer. These OCs are among the most toxic of the PCBs, together with non-*ortho* PCBs. The relatively flat (or “planar”) orientation of their biphenyl rings allows them to bind to the aryl hydrocarbon (Ah) receptor in a similar way to polychlorinated dibenzodioxins and dibenzofurans and may be responsible for our finding (Safe 1990). This binding induces phase I and phase II metabolic enzymes; mono-*ortho* PCBs may therefore induce CYP1A and CYP2B enzymes that, in the absence of substrate, can produce reactive oxygen species. Although PCB-118 has a toxic equivalency factor (TEF) of 0.0001, based on its toxicity relative to 2,3,7,8-tetrachlorinated dibenzo-*p*-dioxin (Van den Berg et al. 1998), PCB-28 has not been assigned a TEF under this scheme and may not, therefore, bind strongly to the Ah receptor and/or significantly induce CYP enzymes. Nevertheless, higher serum levels of PCB-28 were associated with increased risk of colon cancer in this study. Some studies on breast cancer have also reported increased risk being limited to mono-*ortho* PCBs (Aronson et al. 2000; Demers et al. 2002; Lucena et al. 2001).

PCBs themselves are hydroxylated by cytochrome P450 enzymes, and their metabolites have been shown to induce DNA adducts *in vitro* (McLean et al. 1996). We have not observed a clear differential risk for PCB-28 or PCB-118 in relation to K *ras* or *p53* mutations. However, when mutations in both genes were classified according to their molecular nature, an association between transversions (less likely to occur spontaneously) and exposure to mono-*ortho* PCBs was observed. This could be interpreted as indirect evidence for a molecular fingerprint associated with exposure to these pollutants.

The role of other OCs in colorectal cancer risk may be more complex. Compounds such as *p*,*p*′-DDE and α-HCH that had an overall moderate association with colorectal cancer showed a significant increase in risk for tumors with mutation of the *p53* gene. A similar result was reported in a Danish study, where higher risk of breast cancer with mutated *p53* was observed among women exposed to high levels of dieldrin and PCBs (Hoyer et al. 2002). In our population, *p*,*p*′-DDE also increased risk for tumors with wild-type K-*ras* but not when this oncogene was mutated. Previous studies in pancreatic cancer have provided mixed results regarding OC exposure and K-*ras* mutation status associated with DDE levels (Porta et al. 1999; Slebos et al. 2000).

Several factors suggest that these findings may be causal: the strength and trend of the associations observed; the restriction of the findings to mono-*ortho* PCBs that have a relatively planar, dioxin-like structure; the existence of similar findings in tumors from other organs; the plausibility of carcinogenic mechanisms; and the association with specific mutation types. We are aware, however, that this study also has several limitations. Owing to the limitations of our analytical method, we did not study non-*ortho* PCBs that have a greater affinity for the Ah receptor than do mono-*ortho* PCBs as a result of their “coplanar” molecular structure, which may have strengthened our hypothesis. The retrospective assessment of exposure in the case–control design is not ideal, and the use of hospital controls may introduce information and selection bias. We minimized these biases by careful design, the use of validated questionnaires administered by trained interviewers, and robust analytical methods. However, potential sources of non-differential bias would tend to decrease the magnitude of any positive relationship; in particular, if OC exposure increased the risk of hospital admission, this would bias the associations toward the null hypothesis. Furthermore, potential confounding factors were explored in detail and rejected. Special effort was devoted to explore whether the weight loss observed among cases could explain the results, because OCs accumulate in lipids, and changes in BMI due to metabolism of adipose tissue in cancer patients could result in higher serum levels of OCs. However, three observations combine to negate this possibility: *a*) adjusting for BMI had only a minor effect on risk estimates; *b*) although more advanced cases exhibited severe weight loss, there was no association between OC levels and tumor stage; and *c*) if lipid mobilization was the reason for observing higher OC levels among cases, this would be true for all OCs but especially the more lipophilic compounds, whereas we observed increased risk only for selected OCs, and these were less lipophilic than *p*,*p*′-DDT and PCB-138, PCB-153, and PCB-180, for which no increased risk was observed. Nevertheless, we cannot exclude chance as an explanation of some of the results because this study was conceived as exploratory or hypothesis generating and we performed many statistical comparisons.

In conclusion, these results suggest that exposure to mono-*ortho* PCBs is associated with an increased risk of colorectal cancer. The trend and magnitude of the association and the specific toxic action of these PCBs, linked with transversion-type mutations, suggest that this finding may be causal. This hypothesis merits confirmation in other studies that would benefit from including other mono-*ortho* PCBs, as well as non-*ortho* PCBs and perhaps dioxins/furans.

## Figures and Tables

**Table 1:** Population characteristics.

	Controls No. (%)	Cases No. (%)	OR (95% CI)	*p*-Value*^a^*
Sex				
Male	43 (57)	75 (57)	1.00	
Female	33 (43)	57 (43)	1.07 (0.60–1.92)	
Age (years)				
24–63	26 (34)	46 (35)	1.00	
63–73	25 (33)	47 (36)	1.09 (0.54–2.20)	
73–92	25 (33)	39 (30)	0.91 (0.45–1.83)	
Energy intake (calories/day)				
663–1,733	24 (32)	46 (35)	1.00	
1,733–2,280	26 (34)	43 (33)	0.82 (0.39–1.73)	
2,280–4,418	26 (34)	43 (33)	0.83 (0.38–1.82)	
BMI				
16.8–25.0	24 (32)	56 (42)	1.00	0.09
25.0–30.0	33 (43)	51 (39)	0.65 (0.34–1.25)	
30.0–40.7	19 (25)	25 (19)	0.53 (0.24–1.17)	
BMI 10 years before diagnosis				
17.6–25.0	19 (25)	42 (32)	1.00	0.79
25.0–30.0	42 (55)	57 (43)	0.61 (0.31–1.20)	
30.0–43.1	15 (20)	33 (25)	0.95 (0.41–2.18)	
Plasma lipids (g/L)				
2.58–4.94	26 (34)	44 (33)	1.00	0.49
4.94–5.85	21 (28)	48 (36)	1.37 (0.67–2.81)	
5.85–9.55	29 (38)	40 (30)	0.79 (0.39–1.57)	
Alcohol duration (years)				
0	34 (45)	42 (32)	1.00	0.05
1–40	19 (25)	38 (29)	2.22 (0.93–5.30)	
40–74	23 (30)	52 (39)	2.40 (1.02–5.63)	
Alcohol consumption (g/day)				
0	36 (47)	42 (31)	1.00	0.001
1–60	35 (46)	66 (50)	2.42 (1.13–5.17)	
60–410	5 (6)	24 (18)	7.39 (2.13–25.7)	
Tobacco use (men*^b^*)				
Nonsmoker	9 (20)	20 (26)	1.00	0.47
Ex-smoker	23 (53)	37 (49)	0.75 (0.29–2.00)	
Smoker	11 (25)	18 (24)	0.66 (0.21–2.12)	
Smoking duration (years, men*^b^*)				
0	9 (21)	20 (27)	1.00	0.81
1–40	20 (47)	22 (29)	0.48 (0.17–1.36)	
40–79	14 (33)	33 (44)	1.05 (0.38–2.96)	
Parity (women)				
0–2	6 (18)	16 (28)	1.00	0.78
2–4	20 (61)	25 (44)	0.55 (0.17–1.76)	
4–9	7 (21)	16 (28)	1.25 (0.31–5.11)	
NSAID use				
None	52 (68)	101 (76)	1.00	0.12
Aspirin	12 (15)	22 (16)	0.92 (0.42–2.04)	
Other	12 (15)	9 (6)	0.43 (0.16–1.11)	

a Test for linear trend adjusted for age, sex, energy intake, and BMI.

b Only 6% of women ever smoked in this population.

**Table 2:** Lipid-corrected serum concentrations of OCs.

OC	Median*^a^* (cutpoints)*^b^*	LOD*^c^*	Percent of samples > LOD*^d^*
*p,p*′-DDE	3,686 (2,574–5,565)	0.2	100
*p,p*′-DDT	444 (336–740)	1.5	100
HCB	1,753 (1,344–2,522)	77.9	98
α-HCH	17 (< 2.8–30)	2.8	61
β-HCH	1,057 (779–1,422)	34.4	100
γ-HCH	10 (< 1.5–46)	1.5	56
PCB-28	< 3.5 (< 3.5)	3.5	25
PCB-118	92 (< 21.0–127)	21.0	62
PCB-52	< 4.6 (< 4.6)	4.6	9
PCB-153	362 (255–487)	16.0	100
PCB-138	308 (245–404)	15.3	98
PCB-180	252 (190–333)	16.0	98

a Lipid-corrected concentrations expressed as ng/g lipid.

b Cutpoints were 33rd and 66th percentiles, except for compounds for which the 33rd percentile was < LOD, in which case median of measured values were used.

c Lipid corrected (ng/g lipid) using average lipid concentration in the population (5.44 g lipid/L serum).

d Proportion of samples with values > LOD.

**Table 3:** Lipid-corrected serum concentrations [median (5th–95th percentile); ng/g lipid] of OCs in cases and controls.

OC	Cases	Controls
*p,p*′-DDE	3,936 (600–11,804)	2,977 (611–13,608)
*p,p*′-DDT	396 (124–2,077)	609 (137–3,848)
HCB	1,753 (487–5,777)	1,763 (720–4,848)
α-HCH	21 (< 2.8–94)	14 (< 2.8–55)
β-HCH	1,042 (315–2,971)	1,119 (345–3,654)
γ-HCH	10 (< 1.5–121)	10 (< 1.5–399)
PCB-28	< 3.5 (< 3.5–228)	< 3.5 (< 3.5–43)
PCB-118	103 (< 21–319)	63 (< 21–404)
PCB-52	< 4.6 (< 4.6–49)	< 4.6 (< 4.6–< 4.6)
PCB-153	381 (118–1,018)	340 (118–917)
PCB-138	327 (121–992)	301 (91–830)
PCB-180	259 (87–741)	238 (72–650)

**Table 4:** Risk of colorectal cancer associated with organochlorine body burden.

	Controls No. (%)	Cases No. (%)	OR (95% CI)	*p*-Value*^a^*
*p,p*′-DDE				
Low	31 (41)	38 (29)	1.00	0.19
Medium	21 (28)	49 (37)	2.17 (1.03–4.54)	
High	24 (32)	45 (34)	1.60 (0.79–3.25)	
*p,p*′-DDT				
Low	25 (33)	44 (33)	1.00	0.12
Medium	18 (24)	52 (39)	1.58 (0.74–3.36)	
High	33 (43)	36 (27)	0.56 (0.27–1.17)	
HCB				
Low	29 (38)	40 (30)	1.00	0.23
Medium	22 (29)	47 (36)	1.72 (0.83–3.54)	
High	25 (33)	45 (34)	1.60 (0.62–4.15)	
α-HCH				
Low	33 (43)	48 (36)	1.00	0.081
Medium	27 (36)	37 (28)	0.99 (0.49–1.98)	
High	16 (21)	47 (36)	2.02 (0.95–4.29)	
β-HCH				
Low	26 (34)	44 (33)	1.00	0.81
Medium	23 (30)	45 (34)	1.14 (0.55–2.36)	
High	27 (36)	43 (33)	0.88 (0.39–2.02)	
γ-HCH				
< LOD	30 (39)	61 (46)	1.00	0.28
Medium	22 (29)	36 (27)	0.79 (0.38–1.61)	
High	24 (32)	35 (27)	0.69 (0.34–1.38)	
PCB-28				
< LOD	65 (86)	91 (69)	1.00	0.006
> LOD	11 (14)	41 (31)	2.75 (1.29–5.83)	
PCB-118				
< LOD	36 (47)	43 (33)	1.00	0.045
Medium	20 (26)	39 (30)	1.63 (0.80–3.31)	
High	20 (26)	50 (38)	2.02 (1.00–4.08)	
PCB-52				
< LOD	69 (91)	120 (91)	1.00	0.77
> LOD	7 (9)	12 (9)	1.16 (0.42–3.19)	
PCB-153				
Low	30 (39)	39 (30)	1.00	0.57
Medium	22 (29)	48 (36)	1.50 (0.74–3.07)	
High	24 (32)	45 (34)	1.22 (0.59–2.52)	
PCB-138				
Low	26 (34)	43 (33)	1.00	0.79
Medium	26 (34)	44 (33)	0.85 (0.41–1.75)	
High	24 (32)	45 (34)	0.90 (0.43–1.90)	
PCB-180				
Low	29 (38)	41 (31)	1.00	0.63
Medium	24 (32)	44 (33)	1.20 (0.59–2.42)	
High	23 (30)	47 (36)	1.19 (0.57–2.49)	

a *p*-Value for trend adjusted for age, sex, energy intake, and BMI.

**Table 5:** Colorectal cancer risk for selected organochlorines in relation to K-*ras* and *p53* mutations.

	Wild-type		Mutated		
	No. (%)	OR (95% CI)	No. (%)	OR (95% CI)	*p*-Value*^a^*
K-*ras*					
*p,p*′-DDE					
Low	16 (20)	1.00	22 (44)	1.00	0.012
Medium	34 (41)	3.51 (1.49–8.24)	15 (30)	1.07 (0.42–2.72)	
High	32 (39)	2.78 (1.21–6.35)	13 (26)	0.72 (0.29–1.81)	
α-HCH					
Low	28 (34)	1.00	20 (40)	1.00	0.55
Medium	27 (33)	1.22 (0.57–2.61)	10 (20)	0.64 (0.25–1.66)	
High	27 (33)	2.16 (0.94–4.97)	20 (40)	1.75 (0.70–4.37)	
PCB-28					
< LOD	56 (68)	1.00	35 (70)	1.00	0.88
> LOD	26 (32)	2.78 (1.24–6.25)	15 (30)	2.83 (1.13–7.06)	
PCB-118					
< LOD	26 (32)	1.00	17 (34)	1.00	0.42
Medium	25 (30)	1.78 (0.81–3.90)	14 (28)	1.35 (0.53–3.43)	
High	31 (38)	2.27 (1.04–4.96)	19 (38)	1.64 (0.67–4.01)	
*p53*					
*p,p*′-DDE					
Low	12 (31)	1.00	11 (19)	1.00	0.047
Medium	16 (41)	2.24 (0.81–6.16)	20 (34)	2.94 (1.11–7.76)	
High	11 (28)	1.09 (0.39–3.05)	28 (47)	3.44 (1.39–8.47)	
α-HCH					
Low	18 (46)	1.00	16 (27)	1.00	0.045
Medium	10 (26)	0.76 (0.29–2.01)	17 (29)	1.39 (0.58–3.35)	
High	11 (28)	1.22 (0.44–3.34)	26 (44)	3.44 (1.40–8.45)	
PCB-28					
< LOD	29 (74)	1.00	44 (75)	1.00	0.98
> LOD	10 (26)	2.16 (0.79–5.91)	15 (25)	2.06 (0.85–5.01)	
PCB-118					
< LOD	14 (36)	1.00	18 (31)	1.00	0.19
Medium	13 (33)	1.72 (0.65–4.53)	13 (22)	1.31 (0.53–3.26)	
High	12 (31)	1.40 (0.52–3.75)	28 (47)	2.79 (1.22–6.37)	

a *p*-Value for interaction between OC and the genetic alteration adjusted for age, sex, energy intake, and BMI; tests for differences in the OR between wild-type and mutated cases.

**Table 6:** Colorectal cancer risk for PCB-118 in relation to K-*ras* and *p53* mutation type.

	Transitions*^a^*		Transversions*^a^*		
	No. (%)	OR (95% CI)	No. (%)	OR (95% CI)	*p*-Value*^b^*
PCB-118					
< LOD	20 (37)	1.00	6 (18)	1.00	0.071
Medium	17 (32)	1.09 (0.28–4.28)	12 (35)	3.29 (0.66–16.4)	
High	17 (32)	1.93 (0.41–9.09)	16 (47)	6.52 (1.16–36.6)	
Trend test *p*-value*^c^*	0.42			0.038					

a Mutations in K-*ras* and *p53* classified as transitions (G:C→A:T) or transversions (G:C→T:A).

b *p*-Value for interaction between PCB-118 and the mutation type; tests for differences in the OR between transitions and transversions.

c Trend test *p*-value for the specific mutation type adjusted for age, sex, energy intake, and BMI.
